# Foodborne TiO_2_ Nanoparticles Induced More Severe Hepatotoxicity in Fructose-Induced Metabolic Syndrome Mice via Exacerbating Oxidative Stress-Mediated Intestinal Barrier Damage

**DOI:** 10.3390/foods10050986

**Published:** 2021-04-30

**Authors:** Yu Zhao, Yizhou Tang, Shanji Liu, Tiantian Jia, Donggen Zhou, Hengyi Xu

**Affiliations:** 1 State Key Laboratory of Food Science and Technology, Nanchang University, Nanchang 330047, China; 5603515014@email.ncu.edu.cn (Y.Z.); 402313318102@email.ncu.edu.cn (Y.T.); 412314919032@email.ncu.edu.cn (S.L.); 7901119160@email.ncu.edu.cn (T.J.); 2 Ningbo International Travel HealthCare Center, Ningbo 315012, China

**Keywords:** titanium dioxide nanoparticles, fructose, metabolic syndrome, hepatotoxicity, intestinal barrier

## Abstract

The hazard of titanium dioxide nanoparticles (TiO_2_ NPs) in diseased population should be given focus due to the huge number of these NPs in foods and medicine. This study aimed to evaluate the stronger biological adverse effect of oral exposure to TiO_2_ NPs in a fructose-induced metabolic syndrome mouse model. Compared to the normal mice, low-dose (2 mg/kg) TiO_2_ NPs did not cause severe hepatotoxicity. However, high-dose (20 mg/kg) TiO_2_ NPs induced aggravated hepatic inflammation, fibrosis, and apoptosis, with substantial alteration of related biochemical parameters in the mouse model. Moreover, significantly increased Ti and lipopolysaccharide burden were observed in metabolic syndrome murine liver and serum, which possibly worsened the portend intestinal leakage. The expression of tight junction-related protein showed that TiO_2_ NPs induced further increase in serious intestinal permeability. The intestinal inflammatory and oxidative stress response in the model were also assessed. Results showed that TiO_2_ NPs caused more severe intestinal inflammatory injury by intensifying the oxidative stress in metabolic syndrome mice and then induced further liver injury. This work provides information on the insights into the toxic effect of TiO_2_ NPs in sub-healthy population.

## 1. Introduction

Titanium dioxide nanoparticles (TiO_2_ NPs) play an integral role in today’s industry due to their remarkable physicochemical characteristics [1,2]. TiO_2_ NPs have been applied in many fields, including food, cosmetics and paints, that people were forced to live in an environment with everyday contact with TiO_2_ NPs [3]. As a food addictive (E171), 36–40% of TiO_2_ particles exist under a nanoscale form [4,5]. Based on nanoparticle intake data, children ingest approximately 5 mg/kg BW TiO_2_ NPs per day, while adults ingest 1–2 mg/kg BW per day [6]. Although TiO_2_ NPs are considered safe, recent studies have found that TiO_2_ NPs could increase intestinal permeability, which leads to NPs crossing the intestinal barrier more easily and reaching in other organs, such as liver [7,8]. Then, TiO_2_ NPs could induce oxidative stress, inflammatory cell infiltration, cell apoptosis, and DNA damage in the liver of a healthy body [9,10], however, the pathological characteristics are not serious or obvious enough to develop into a hepatic disease. Some results even showed that a mild dosage of TiO_2_ NPs is not toxic to a healthy body [11,12].

However, due to the TiO_2_ NPs being added in foods and medicines, the disease population possibly received a higher amount of TiO_2_ NPs than normal. A study found that TiO_2_ NPs could aggravate colon injury in DSS-induced colitis mice [13], and TiO_2_ NPs were proven to be an adjuvant in allergic airway diseases. [14]. These findings demonstrated that TiO_2_ NPs may cause worsened outcomes in patients.

Metabolic syndrome is a pathological condition that is due to imbalanced carbohydrate, protein, fat, and other substances metabolized in the human body. The global prevalence of many diseases related to metabolic syndrome is rising. These diseases include nonalcoholic fatty liver disease (NAFLD), type 2 diabetes, hypertension, and even cancer. According to reports, 20–30% of the adult population could be characterized as suffering from metabolic syndrome, and the global prevalence of NAFLD is approximately 24% [15]. Increased fructose consumption may be a major reason for the increasing trend in the prevalence of NAFLD. Fructose with 22% of five-membered furanose ring in a solution not only could elevate de novo lipogenesis, triglyceride formation, and steatosis but also promote production of reactive oxygen species (ROS) due to its unstable structure [16]. These two functions of fructose correspond with the mechanism theory of NAFLD [17]. TiO_2_ NPs were notably widely used in sugary products and drug carriers [3], a number of TiO_2_ NPs accompanied by fructose or medicine could be taken up by patients with metabolic syndrome-related diseases. Thus, increased attention should be paid to the risk of TiO_2_ NPs exposure in this population. Moreover, the liver is a target organ of TiO_2_ NPs, which induce excessive ROS. Therefore, evaluating the effect of TiO_2_ NP exposure in populations with metabolic syndrome is significative. Meanwhile, an increase in intestinal permeability was observed in patients with NAFLD [18], and the negative effect of TiO_2_ NPs to the intestinal barrier was commonly observed in cells and animals [19,20]. Worsening of intestinal barrier damage may cause increased nanoparticles in the liver, thereby exacerbating hepatic injury. Thus, we speculated TiO_2_ NPs could worsen the hepatic injury in a metabolic syndrome model by attacking the intestinal barrier.

In this study, we aimed to evaluate whether TiO_2_ NPs could cause stronger adverse effects in the fructose-induced metabolic syndrome mouse model. More importantly, the mechanism by which TiO_2_ NPs aggravate liver injury by intensifying intestinal barrier damage was explored. This study could provide insights into TiO_2_ NPs application in sub-healthy population, such as those with metabolic syndrome.

## 2. Materials and Methods

### 2.1. TiO_2_ Nanoparticles

TiO_2_ NPs (CAS 13463-67-7 10–25 nm) was purchased from Aladdin industrial Corporation, Shanghai, China. TiO_2_ NPs used in this work were characterized in our previous study [21], with a spherical distribution and their diameter was 25.20 nm, it was anatase crystals, which were closer to food products. The hydrodynamic size of nanoparticles is 88.28 ± 20.31 nm in pH 1, 112.05 ± 33.64 nm in pH 5, and 138.41 ± 45.51 nm in pH 7. The nanoparticle dispersibility was great in the environment of acidic fluid.

### 2.2. Animal Experimental Procedure

Fifty healthy male Kunming mice (5 weeks old) were obtained from the experimental animal center of Nanchang University, China. The mice were placed in rooms with a 12 h light and 12 h dark cycle, the temperature and relative humidity were 23 ± 2 °C and 50 ± 5%. Every animal procedure in this study was guided under the institutional animal care committee guidelines and approved by the Animal Care Review Committee (approval number 0064257), Nanchang University, Jiangxi, China. After 1 week of acclimatization, mice were randomly divided into five group (*n* = 8~10), Respectively, Group I mice were ingested ordinary water by drinking as the control group. Group II mice were ingested 20 mg/kg BW (body weight) TiO_2_ NPs with ordinary drinking water (20 mg/kg). Group III, mice were feeding 30% (wt/vol) fructose (Fru). Group IV, mice were treated with 2 mg/kg BW TiO_2_ NPs and 30% fructose (2 mg/kg + Fru). Group V, mice were treated with 20 mg/kg BW TiO_2_ NPs and 30% fructose (20 mg/kg + Fru). The TiO_2_ NPs were dispersed in 1×PBS solution, ultrasonicated for 30 min and vortex scattered before gavage. Fructose was fed by adding to the ordinary drinking water, and TiO_2_ NPs were ingested by orally administrated once a day. The low dosage of the TiO_2_ NPs (2 mg/kg) in this work is refer to the actual level of human exposure, and the high dosage (20 mg/kg) is a tenfold increasing. The body weight and waistline of mice were recorded during the experiment.

After exposure to TiO_2_ NPs or fructose for 8 weeks, mice were euthanized and the blood, liver, intestine and epididymis fat were isolated. Blood was collected by eyeball removal and put into an enzyme-free and sterile centrifuge tube, which was then placed in a 4 °C environment. Six hours later, serum was centrifuged at 4000 rpm for 10 min at 4 °C and stored at −80 °C immediately for subsequent studies. The liver and epididymis fat tissue were weighed to calculate the organ coefficient (the organ weight divide by the mice body weight). Then, liver and intestine were collected and preserved in the −80°C freezer immediately for subsequent analysis.

### 2.3. Oral Glucose Tolerance Test (OGTT)

The OGTT was performed after 54 days exposure. Mice were fasted for 12 h before the experiment. Then, collected the tail venous blood and measured fasting blood-glucose immediately. After that, four mice were randomly selected from each group and oral administration of 2 g/kg glucose solution. Blood glucose levels were measured at 15, 30, 60, 90, 120 min by tail vein (Cofoe glucose meter. Qingdao, China).

### 2.4. Serum Biochemical and Liver Lipid Assays

Serum alanine aminotransferase (ALT), aspartate aminotransferase (AST), alkaline phosphatase (ALP), cholesterol and triglycerides were measured by using a commercial kit (Jiancheng Bioengineering Institute. Nanjing, China). All the processes were according to the manufacturer’s instructions. Liver tissue were homogenized in normal saline (10% w/v) and centrifuged at 3000× *g* for 15 min at 4 °C, the supernatant was used for measuring the level of cholesterol and triglycerides as above method.

### 2.5. Histopathological Analysis

The fresh liver and intestine were fixed in 4% paraformaldehyde, dehydrated with different concentrations of ethanol and embedded in a paraffin cube, then sliced to 4–5 µm thick sections (*n* = 3 for each group). Liver slides were performed H&E staining and Masson staining, while intestine made H&E staining. Using a Nikon Tι optical microscope (Tokyo, Japan) to gain each section’s image for histologic evaluations. As for liver, the pathology was evaluated by H&E staining and the fibrosis was by Masson staining. Inflammations were evaluated (0, no inflammatory cell. 1, slight portal inflammation. 2, mild portal and local tissue inflammatory infiltration. 3, extensive portal and tissue inflammatory infiltration). The ratio of Masson blue^+^ area was quantitated by Image J. As for intestine, the epithelial injury scores were evaluated (0, no structure damage. 1, mild villi hypertrophy and crypt disorder. 2, villi hypertrophy and crypt disorder, and slight lamina propria attenuation. 3, distinct villi hypertrophy and crypt disorder, lamina propria and intestinal wall damage). The inflammation was evaluated by scoring with indices (0, no inflammatory infiltration. 1, slight infiltration in lamina propria. 2, mild infiltration and spread to the crypt. 3, extensive infiltration and permeated to muscular layer).

### 2.6. Ti Content Detection

After 8-week TiO_2_ NPs gavage, five mice’s liver per group from 20 mg/kg and 20 mg/kg + Fru group were collected to determinate Ti contents. In brief, 0.4–0.5 g of liver was put into an acid solute on of HNO_3_ and HClO_4_ (10 mL and 2 mL), and then heated to 280 °C until the solution become clear. When the nitrate solution was dried, resuspended with the ultrapure water and transferred to a new glass cube (unified to 25 mL), an inductively coupled plasma atomic emission spectrometer (ICP-AES Optima 8000. PerkinElmer Inc., Waltham, MA, USA) was used to detect Ti contents.

### 2.7. ELISA

Liver and intestine homogenate (10% w/v) were prepared as the part of 2.4. Both supernatant of liver and intestine were used for measuring tumor necrosis factor α (TNF-α), Interleukin (IL) 1β, IL 6, and IL 10. The level of lipopolysaccharide (LPS) in serum and liver were evaluated. The above biochemical factors were tested by ELISA in accordance with the manufacturer’s description (EYKITS Co., LTD., Shanghai, China).

### 2.8. Oxidative Stress Assay

The hepatic and intestinal oxidative stress of mice were ascertained by testing of superoxide dismutase (SOD), catalase (CAT) as well as glutathione (GSH) activity, malondialdehyde (MDA) level. Liver and intestine homogenate (10% *w*/*v*) were used for the various estimations. The above biochemical parameters were testing by the commercial kit (Jiancheng Bioengineering Institute. Nanjing, China). All the processes were according to the manufacturer’s instructor.

### 2.9. RT-qPCR

The RNA of the liver and intestine from each group was extracted using AxyPrep Multisource Total RNA Miniprep Kit (Axygen Scientific, CA, USA). The same amount of RNA was reverse transcribed to cDNA by using Takara PrimeScript TM RT reagent kit (cat. no. RR047A) after evaluated the concentration of total RNA by NanoDrop 1000 spectrophotometer (Thermo scientific Inc, US). Quantitative PCR (qPCR) was performed with TB Green™ Premix Ex Taq™ II (TIi RNaseH Plus, TAKARA Cat#RR820A) on the AriaMx Real-Time PCR System (Agilent, Inc. CA, US). Following cycle program involved 1 cycle at 95 °C for 60 s, then 40 cycles of 95 °C for 5 s, then 59 °C for 60 s, 72 °C for 30 s. The primers were showed in Table 1, and they were synthesized by TSINGKE Biological Technology (Beijing, China).

### 2.10. Immunohistochemistry

The intestine was dewaxed and washed with PBS, transferred to 3% H_2_O_2_ for 25 min to prevent endogenous peroxidase, rinsed, and incubated in a blocking solution (3% TBS) for 30 min at room temperature. Sections were incubated with primary antibodies against ZO-1 and Occludins (OCLN) (1:500) overnight at 4 °C, rinsed, incubated in secondary antibodies-HRP (Servicebio, GB23303 Wuhan, China) for 50 min at room temperature, developed with diaminobenzidine (DAB). immunohistochemistry (IHC) score was evaluated by Image J (National Institutes of Health, Bethesda, MD, USA). A, the immunostaining intensity (negative = 0, low positive = 1, positive = 2, high positive = 3). B, percentage of immunostaining area (0–25% = 1, 26–50% = 2, 51–75% = 3, 76–100% = 4). The final score was the sum of A and B.

### 2.11. Statistical Analysis

All the data in this work were expressed as means ± SD. Using the SPSS v22.0 (SPSS, Inc., Chicago, IL, USA) to perform one-way analysis of variance (ANOVA) for comparing the results between the different groups. 2^−ΔΔCt^ method was used to analyze the qPCR results. *P* < 0.05 was considered as significance.

## 3. Results

### 3.1. TiO_2_ NPs Does Not Affect Fructose-Induced Metabolic Syndrome Formation

**Figure 1:**
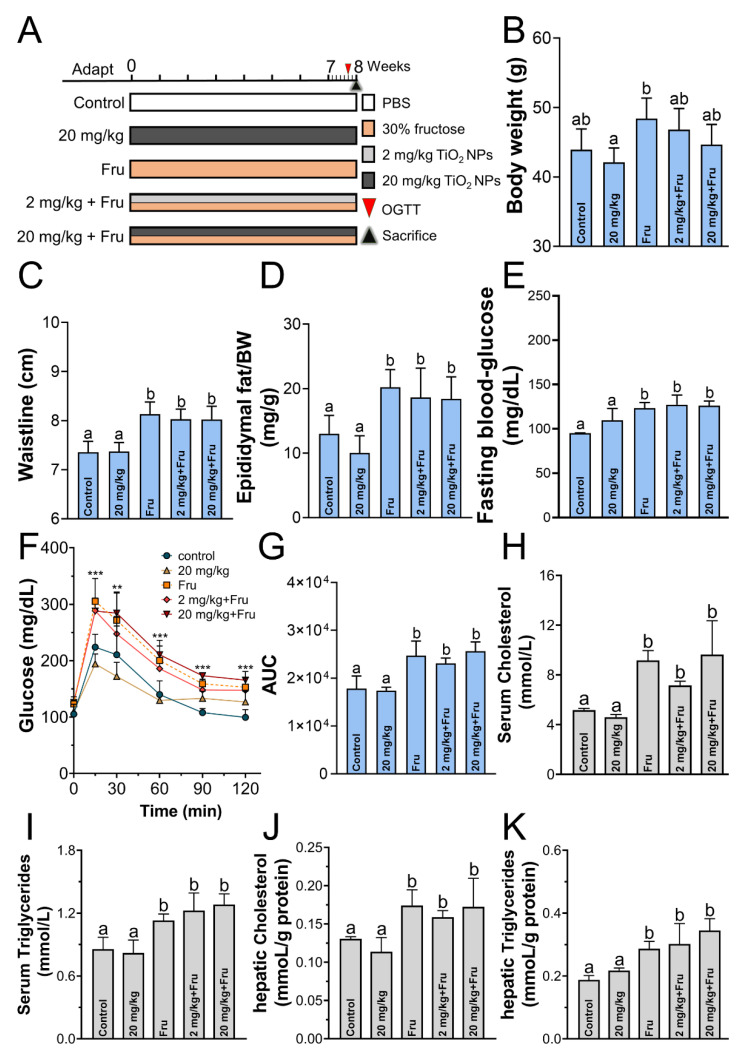
Fructose could induce metabolic syndrome after experiment. (**A**) Experimental design. (**B**–**D**) Murine body weight, waistline, epididymal fat ratio, fasting blood-glucose after 8-weeks exposure. (**F**) Blood glucose level in OGTT (*n* = 4 mice/group). (**G**) Glucose area under the curve (AUC). (**H**,**I**) The concentration of cholesterol and triglycerides in serum. (**J**,**K**) The concentration of cholesterol and triglycerides in liver. *n* = 8~10. One-way ANOVA, different superscript alphabets represent significance between each group and ** *p* < 0.01, *** *p* < 0.001, versus the control group.

The experimental design was showed in Figure 1A. After treatment with fructose for 8 weeks, their body weights (BW) did not obviously increase compared with the control (Figure 1B), whereas their murine waistline, epididymal fat/BW, and fasting blood glucose significantly increased (Figure 1C–E). In addition, the mice showed remarkable weakness in glucose tolerance (Figure 1F,G). A significant increase in the cholesterol and triglyceride levels in the serum and liver was also observed (Figure 1H–K). Therefore, the metabolic syndrome model was successfully established. The above physical parameters did not differ between the fructose-only group and TiO_2_ NPs groups with fructose-exposure (Figure 1).

### 3.2. TiO_2_ NPs Aggravated Liver Inflammation Injury in Metabolic Syndrome Mice

**Figure 2:**
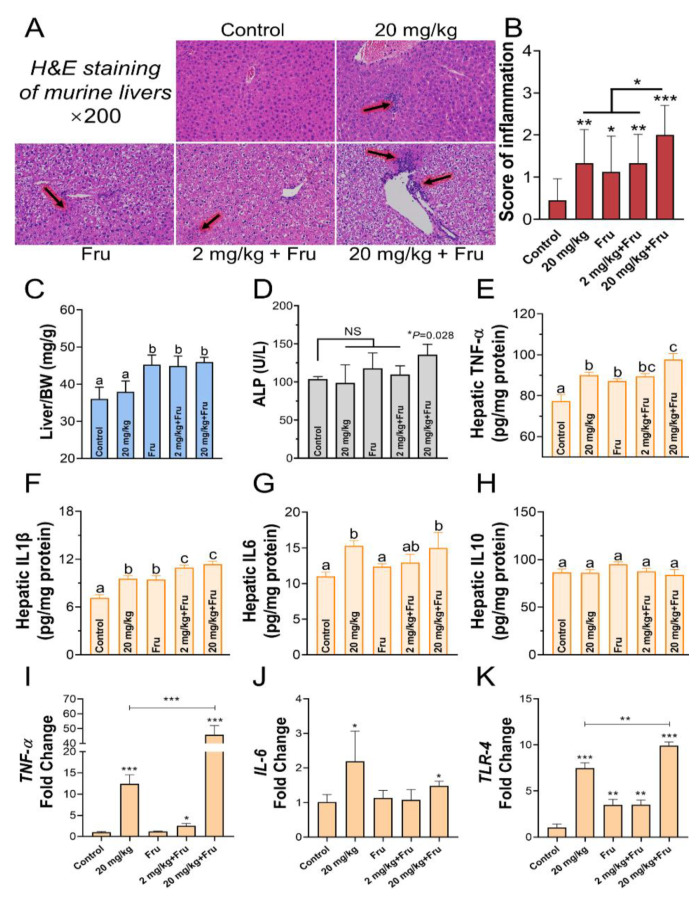
TiO_2_ NPs aggravated hepatic inflammatory injury in metabolic syndrome mice. (**A**) H&E staining of murine livers, the black luminous arrow indicated inflammatory cell infiltration. (**B**) the score of inflammation for H&E staining of livers (every staining slide randomly selected three different views to evaluated scores). (**C**) Hepatic viscera coefficient, liver (mg) divide body weight (g) (*n* = 8~10 mice/group). (**D**) the level of alkaline phosphatase (ALP) in serum. (**E**–**H**) The concentration of tumor necrosis factor α (TNF-α), Interleukin (IL) 1β, IL 6, and IL 10 in liver. (**I**–**K**) the mRNA expression levels of *TNF-α*, *IL 6*, and *TLR-4* in liver. *n* = 5/ group. One-way ANOVA, different superscript alphabets represent significance between each group and * *p* < 0.05, ** *p* < 0.01, *** *p* < 0.001 versus control or between two specific groups.

Hepatocyte swelling and fat accumulation were found in the fructose-exposure groups, while a slight inflammatory infiltration was illustrated by the TiO_2_ NPs group (Figure 2A). The TiO_2_ NPs (20 mg/kg) with fructose-exposure group, exhibited more serious inflammation damage than the fructose-only group (Figure 2B). Compared with the control, after treatment with fructose showed a significant increase in the organ coefficient of liver (Figure 2C). No change was found in the ALT and AST levels of each group (data not shown), but a significant increase in ALP was observed in the fructose-exposure TiO_2_ NPs group (20 mg/kg) (Figure 2D). Next, the concentration of inflammatory factors in the liver was evaluated. TiO_2_ NPs induced an increase in the TNF-α, IL-1β, and IL-6 levels in normal mice. In the fructose-exposure groups, the 20 mg/kg TiO_2_ NPs induced a substantial increase in the TNF-α and IL-1β levels compared with the normal mice and fructose-only group (Figure 2E–G). No change was found in the IL-10 level of each group (Figure 2H). The mRNA expression of the *TNF-α*, *IL6*, and *TLR-4* in the liver was assessed (Figure 2I–K), and the results revealed that the fructose-exposure TiO_2_ NPs groups (20 mg/kg) had the most and dramatical upregulated expression of *TNF-α* and *TLR-4* among the 20 mg/kg group and the fructose-only group.

### 3.3. TiO_2_ NPs Caused Distinct Liver Fibrosis and Apoptosis in Metabolic Syndrome Mice

**Figure 3:**
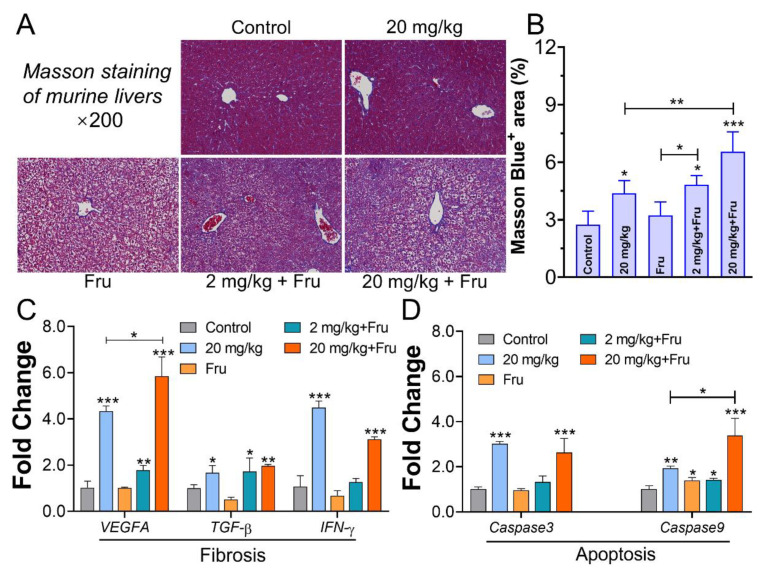
TiO_2_ NPs induced more obvious hepatic fibrosis and apoptosis in metabolic syndrome mice. (**A**) Masson staining of murine livers. (**B**) The quantities of Masson blue area ratio (every staining slide randomly selected three different views to evaluated scores). (**C**) The mRNA expression level of fibrosis factors in liver. (**D**) The mRNA expression level of apoptosis factors in liver. *n* = 5/group. One-way ANOVA, * *p* < 0.05, ** *p* < 0.01, *** *p* < 0.001 versus control or between two specific groups.

The Masson staining image illustrated fibrosis in murine liver from the different groups (Figure 3A). The TiO_2_ NP group showed a significant increase in Masson blue^+^ ratio compared with the control. Specially, in the combination of fructose and TiO_2_ NP treatment groups, 20 mg/kg TiO_2_ NPs induced the largest ratio among others (Figure 3B). Subsequently, the expression of fibrosis-related genes in the liver was detected (Figure 3C). TiO_2_ NPs increased fibrosis-related gene expression in the normal mice, with a significant upregulation in *vascular endothelial growth factor A* (*VEGFA*), *transforming growth factor-β* (*TGF-β*), and *interferon gamma* (*IFN-γ*) expression. In the fructose-exposure groups, the 2 mg/kg TiO_2_ NPs group showed an upregulation of *VEGFA* and *TGF-β*, while the 20 mg/kg TiO_2_ NPs group showed a more intensive expression of *VEGFA* than the normal mice. The apoptosis-related genes were also investigated. A remarkable increase in the expression of *Caspase 3* and *Caspase 9* was observed after exposure to TiO_2_ NPs in normal mice. Among the fructose-exposure groups, the 20 mg/kg TiO_2_ NPs group exhibited the most significantly upregulated *Caspase 3* and *Caspase 9*, whereas the fructose-only group only showed increased *Caspase 9* (Figure 3D).

### 3.4. TiO_2_ NPs Exacerbated Liver Oxidative Stress in the Metabolic Syndrome Mice 

**Figure 4:**
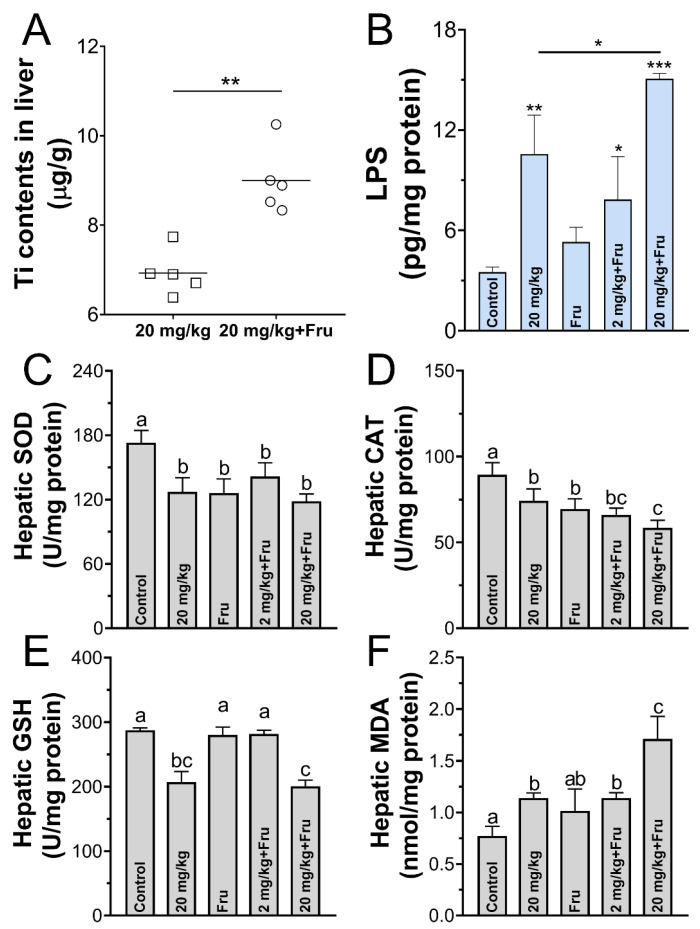
TiO_2_ NPs induced intensified oxidative stress in metabolic syndrome mice. (**A**) The amounts of Ti element in liver. (**B**) The concentration of lipopolysaccharide (LPS) in liver. (**C**–**E**) The activity of the SOD, CAT, GSH in liver. (**F**) The level of the MDA in the liver. *n* = 5/group. One-way ANOVA, different superscript alphabets represent significance between each group and * *p* < 0.05, ** *p* < 0.01, *** *p* < 0.001 versus control or between two specific groups.

The results showed a significantly larger amount of Ti and LPS in the fructose-exposure group than in the normal mice after exposure to TiO_2_ NPs, whereas the fructose-only group showed no differences in hepatic LPS level compared with the control (Figure 4A,B). Next, the oxidative stress in liver was assessed. Compared with the control, TiO_2_ NPs lead a considerable decrease in the SOD, CAT, and GSH activities in normal mice. However, among the fructose-exposure groups, the 20 mg/kg TiO_2_ NPs group showed the lowest SOD, CAT, and GSH levels, whereas the fructose-only group demonstrated a decrease in the SOD and CAT levels only (Figure 4C–E). On the contrary, the combination of fructose and TiO_2_ NPs (especially 20 mg/kg) treatments remarkably increased the concentration of MDA (Figure 4F).

### 3.5. TiO_2_ NPs Aggravated Intestinal Permeability Increase in Metabolic Syndrome Mice 

**Figure 5:**
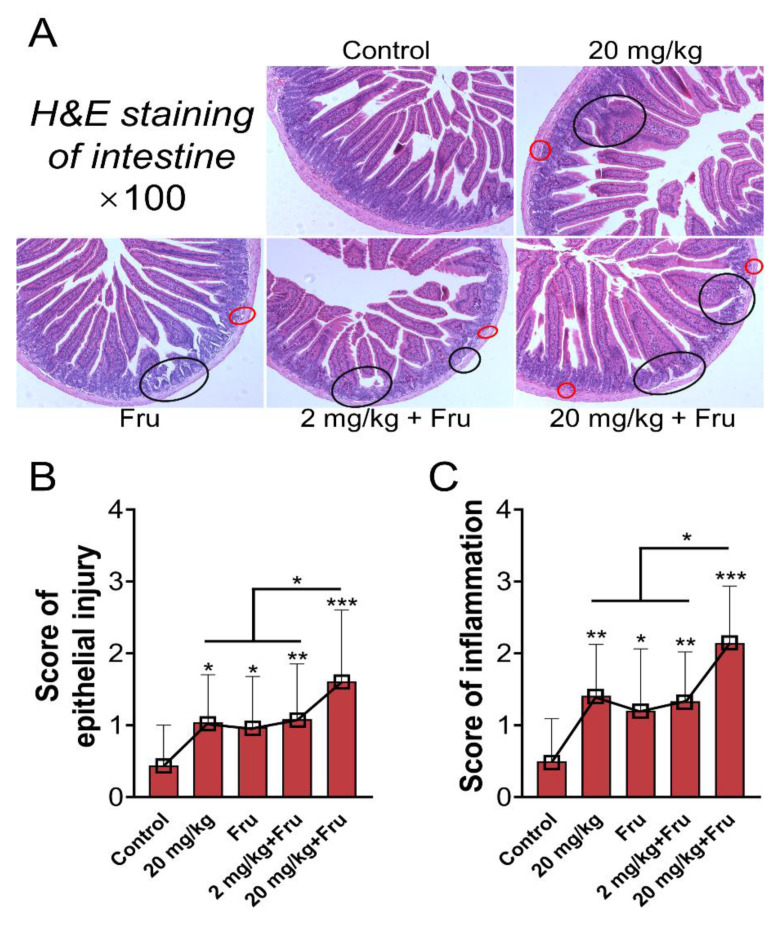
TiO_2_ NPs caused more serious intestinal structure damage in metabolic syndrome mice. (**A**) The H&E staining of intestine, the black circle indicated local mucous lamina propria is disordered, the red circle distinguished inflammatory infiltration. (**B**,**C**) The score of epithelial injury and inflammation (every staining slide randomly selected three different views to evaluated scores). *n* = 5/group. One-way ANOVA, * *p* < 0.05, ** *p* < 0.01, *** *p* < 0.001 versus control or between two specific groups.

**Figure 6:**
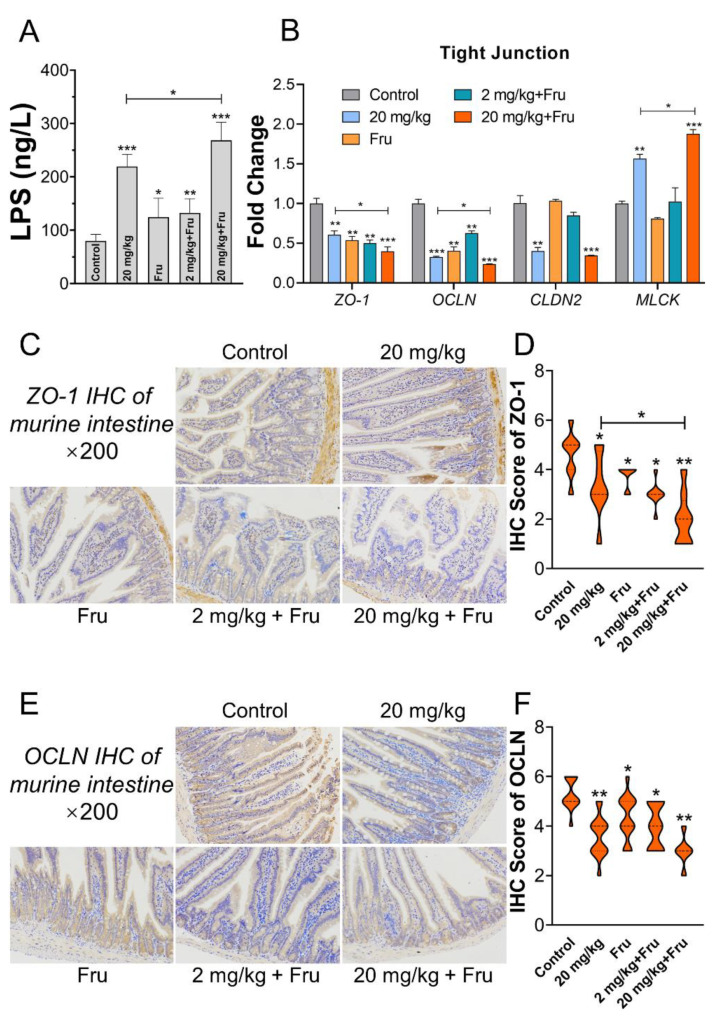
TiO_2_ NPs induced more obvious intestinal permeability increase in metabolic syndrome mice. (**A**) The level of LPS in serum. (**B**) the mRNA expression level of tight junction (TJ)-related protein in the intestine. (**C**) ZO-1 immunohistochemical staining of murine intestine. (**D**) The score of ZO-1 immunohistochemical staining (every staining slide randomly selected three different views to evaluated scores). (**E**) OCLN immunohistochemical staining of murine intestine. (**F**) The score of OCLN immunohistochemical staining (every staining slide randomly selected three different views to evaluated scores). *n* = 5/group. One-way ANOVA, * *p* < 0.05, ** *p* < 0.01, *** *p* < 0.001 versus control or between two specific groups.

Histological analysis of the intestine revealed that TiO_2_ NPs exposure could lead to villi hypertrophy and crypt disorder in normal and fructose-exposure mice. Reduced inflammatory infiltration and lamina propria mucosae were discovered in all of treatment groups (Figure 5A). The scores of epithelial injury and inflammation are shown in Figure 5B,C. Compared with the control, the experimental groups showed a significant increase in score after exposure to TiO_2_ NPs or fructose. In particular, the fructose-exposure TiO_2_ NPs group (20 mg/kg) had the highest score. Next, intestinal permeability was evaluated, and the LPS level in serum was considerably increased after exposure to TiO_2_ NPs or fructose (Figure 6A). The combination of 20 mg/kg TiO_2_ NPs and fructose resulted in further increase compared with the normal mice. The expression of tight junction (TJ) target genes in the intestine was further analyzed (Figure 6B), and the results showed that TiO_2_ NPs or single fructose treatment could downregulate the expression of *ZO-1* and *OCLN*. Among the fructose- exposure groups, the 20 mg/kg TiO_2_ NP group demonstrated the lowest expression of *ZO-1*, *OCLN*, and *CLDN2*. In addition, TiO_2_ NPs induced a significant upregulation of *MLCK* in normal mice and a more obvious one in mice after treatment with fructose. Protein expression was further evaluated via immunohistochemistry. Compared with single exposure, 20 mg/kg TiO_2_ NPs with fructose resulted in the lowest ZO-1 and OCLN levels. (Figure 6C–F).

### 3.6. TiO_2_ NPs Induced Drastic Intestinal Inflammation in Metabolic Syndrome Mice

**Figure 7:**
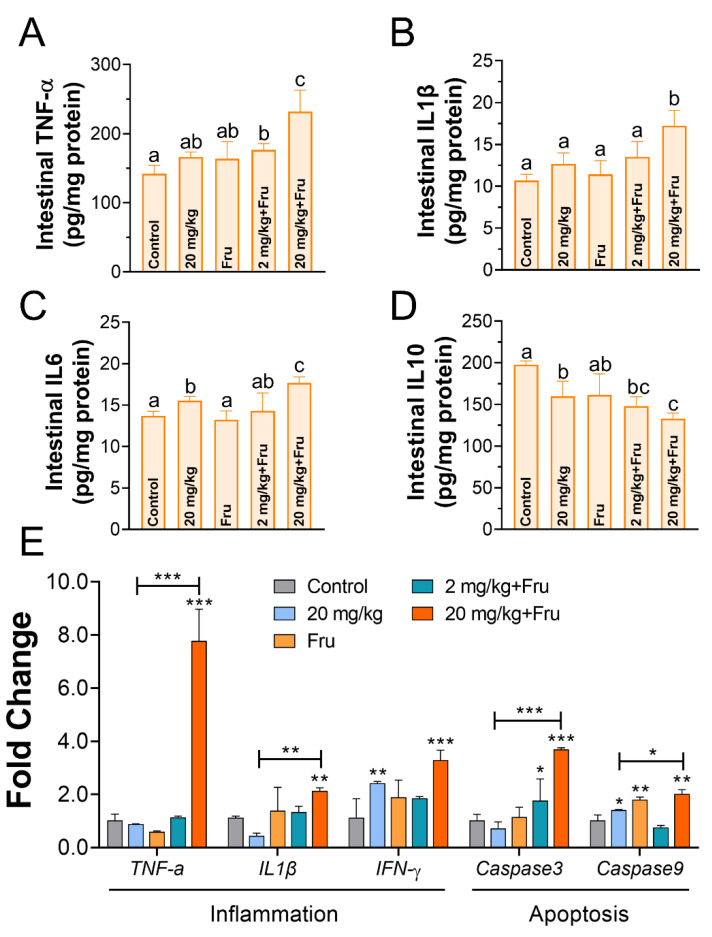
TiO_2_ NPs lead more severe intestinal inflammatory response in metabolic syndrome mice. (**A**–**D**) The concentration of the TNF-α, IL 1β, IL 6, and IL 10 in the intestine. (**E**) The mRNA expression level of inflammatory and apoptosis factors in the intestine. *n* = 5/group. One-way ANOVA, different superscript alphabets represent significance between each group and * *p* < 0.05, ** *p* < 0.01, *** *p* < 0.001 versus control or between two specific groups.

The inflammatory change in the intestine was evaluated. The amounts of TNF-α, IL-1β, IL-6, and IL10 were detected using ELISA (Figure 7A–D). Compared to the control, TiO_2_ NPs just induced evidently increased IL6 level, while decreased IL10 in normal mice. Among the fructose-exposure groups, the fructose single group showed no difference, whereas the TiO_2_ NPs group demonstrated a considerable increase in the TNF-α, IL-1β, and IL-6 levels and a decrease in the IL-10 level. The expression of inflammation-related and apoptosis-related genes was also detected (Figure 7E), compared to the normal mice, A substantial upregulation in the expression levels of *TNF-α*, *IL-1β*, and *IFN-γ* and *Caspase 3* and *Caspase 9* in the fructose-exposure TiO_2_ NPs group (20 mg/kg) was observed compared with that in the normal mice.

### 3.7. TiO_2_ NPs Excavated Intestinal Oxidative Stress in Metabolic Syndrome Mice

**Figure 8:**
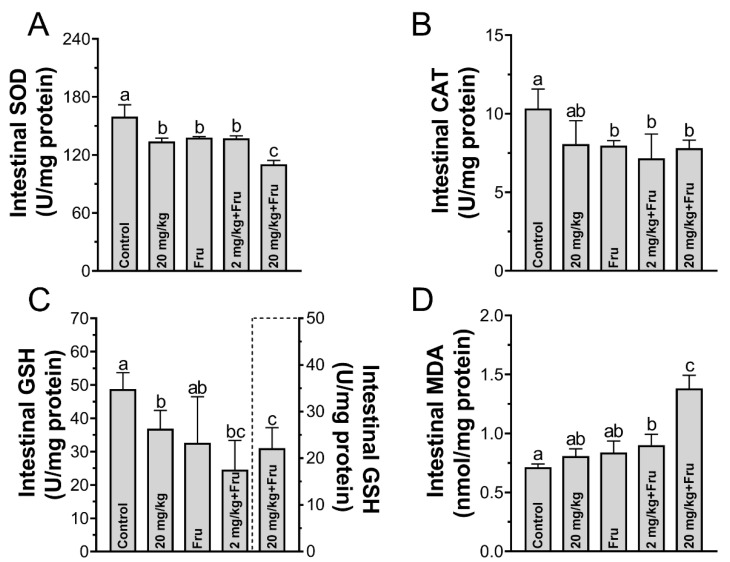
TiO_2_ NPs stimulated stronger oxidative stress in metabolic syndrome mice. (**A**–**C**) the activity of SOD, CAT, and GSH in the intestine. (**D**) the concentration of the MDA in the intestine. *n* = 5/group. One-way ANOVA, different superscript alphabets represent significance between each group.

The oxidative stress in the intestine was evaluated in the different groups (Figure 8A–D). TiO_2_ NPs decreased the SOD and GSH activities in the normal mice compared with those in the control. Among the fructose-exposure groups, the fructose single group exhibited alteration in SOD and CAT, while 2 mg/kg TiO_2_ NPs induced a significant decrease in the GSH activity and a significant increase in the MDA activity compared with the control. More importantly, 20 mg/kg TiO_2_ NPs group showed the most substantial and obvious alteration in the SOD, CAT, GSH, and MDA levels.

## 4. Discussion

According to the definition of the metabolic syndrome and previous study [22], the metabolic syndrome model was built success in our work. No change of the body weight was observed, and this result was consistent with Cho et al. [23] who found that fructose did not result in significant weight gain for mice. Moreover, the body indicators with no difference between fructose-alone group and combine fructose and TiO_2_ NPs group, which manifested that TiO_2_ NPs aggravate liver injury but not due to change in body weight, glucose tolerance, or liver lipid accumulation.

In this work, slight portal vein and tissue inflammatory infiltration was observed in the normal mice after exposure to TiO_2_ NPs, or the metabolic syndrome mice. These results were consistent with those of previous studies [24]. However, no changes in liver coefficient and enzyme activity were found in normal mice after exposure to TiO_2_ NPs, indicating the limited influence of TiO_2_ NPs to the healthy body. These results were similar with Yang et al. and Yao et al. [25,26], The higher level of the inflammatory cytokines of TNF-α, IL1β, IL6, and TLR-4 revealed that high-dose TiO_2_ NPs induced more serious inflammatory liver injury in metabolic syndrome mice.

Persistent or chronic inflammation could lead to fibrosis and apoptosis. Hong et al. [27] fed 10 mg/kg TiO_2_ NPs to mice for 9 months, the hepatic inflammation and fibrosis were observed. In this work, liver fibrosis and apoptosis were investigated. The collagenous fiber was distinguished as blue by Masson staining. No change was observed in the fructose-only group, which indicating that TiO_2_ NPs triggered liver fibrosis but not fructose. *VEGFA*, *TGF-β*, and *IFN-γ* are three classic factors that reflect the pathological development of fibrosis [28]. No difference in gene expression was observed in metabolic syndrome mice. This result was consistent with that of Cho et al. [23], who demonstrated that feeding fructose for 8 weeks did not induce fibrosis in mice. In the present study, TiO_2_ NPs boosted the development of fibrosis in metabolic syndrome mice, and this finding could be explained by the aggravated inflammatory reaction by TiO_2_ NPs. A TiO_2_ NP-exposure safety assessment in the healthy mice was performed by Cui et al. [29], the apoptotic liver cell was turned to observed in the dose of 10 mg/kg and 50 mg/kg. Moreover, TiO_2_ NPs could stimulate liver cell stress response and induce cell oxidative stress [30]. Then, it caused the release of mitochondrial cytochromes c which has regarded as an apoptosis-inducing factor. Cytochromes c could form the apoptosome by binding to the Apaf-1 (apoptotic protease activating factor-1), Caspase 9 precursors and ATP/dATP (adenosine triphosphate/deoxyadenosine triphosphate) then call and activate Caspase 3, thus triggering the caspase cascade reaction, which could degrade the proteins in cell and lead cell apoptosis [31]. In our works, the expression change in *Caspase 3* and *Caspase 9* was evaluated. High-dose TiO_2_ NPs could heighten their expression of in metabolic syndrome mice than normal one, these results are a further proof of the TiO_2_ NP aggravated hepatotoxicity in the metabolic syndrome mice.

Adverse effects in liver after exposure TiO_2_ NPs have been reported in healthy mice. Hong et al. [32] showed 10 mg/kg BW TiO_2_ NPs caused histopathological changes including inflammatory infiltration and local hepatocyte necrosis. Talamini et al. [33] demonstrated repeated administration of 5 mg/kg BW foodborne TiO_2_ NPs could deposit in liver and stimulate molecular and cellular change in the inflammatory respond. As for fructose, which is a major risk factor for the development of metabolic syndrome diseases [16], Bergheim et al. [24] let mice free access to drink containing 30% fructose for 8 weeks, fat accumulation, and lipid peroxidation factors expression were significantly higher. In a word, both the TiO_2_ NPs and fructose would cause mild inflammation and liver dysfunction in normal mice, TiO_2_ NPs are active exogenous chemicals that could stimulate immunological stress in the body, and fructose could accelerate the accumulation of lipids and ROS and cause liver oxidative stress and inflammatory damage [16]. Meanwhile, oxidative stress is a major factor that stimulates inflammation and apoptotic signaling pathways [34]. Studies have also shown that TiO_2_ NPs could cause organ injury by activating oxidative stress [35]. Therefore, the synergetic effect of TiO_2_ NPs and fructose on oxidative stress was evaluated in the present study. This effect was possibly the immediate reason why TiO_2_ NPs intensify the liver damage in metabolic syndrome mice. SOD, CAT, and GSH are three typical antioxidant enzymes, and MDA is the product of lipid oxidation. The fluctuation of their level could reflect body oxidative stress response. As expected, high-dose TiO_2_ NPs led to the most considerable effect of oxidative stress in metabolic syndrome mice than other mice, indicating that metabolic syndrome mice are more susceptible to TiO_2_ NPs. Because the TiO_2_ NPs and LPS levels in metabolic syndrome mice were higher than normal mice under the same dosage of TiO_2_ NPs.

The serum LPS level is known to reflect the change in intestinal permeability. The results of this study suggested that TiO_2_ NPs caused more severe intestinal permeability in metabolic syndrome mice. After oral administration of TiO_2_ NPs, they could damage and pass through the intestinal barrier. Then, they arrive and stay in the organs or tissues and trigger adverse effects. Chen et al. [36] reported that TiO_2_ NPs could induce hepatotoxicity by the indirect pathway of gut-liver axis, such as lead LPS level increased in gut, which as a crucial factor participate in subsequent liver damage. In addition, fructose feeding could promote gut leakage in mice [23]. Therefore, the intestinal barriers were under worsened condition in metabolic syndrome mice after exposure to TiO_2_ NPs in this work.

Then, we further investigated the influence of the intestinal barrier in metabolic syndrome mice after TiO_2_ NPs exposure. The intestinal physical barrier is mainly composed of epithelial cell and its tight junction. The H&E results demonstrated that TiO_2_ NPs induced more obvious luminal epithelium disorder and inflammation in metabolic syndrome mice than in other mice. *ZO-1*, *OCLN*, and *CLDN2* were indispensable to the organization and stability of TJ, and *MLCK* upregulation could cause MLC phosphorylation and actin contraction, which may result in splayed paracellular channel [37]. In the present study, the TiO_2_ NPs or fructose-only groups showed an alteration in the expression of these genes or proteins. This finding was consistent with that of previous studies [23]. Importantly, high-dose TiO_2_ NPs led to more obvious fluctuation of the expression of TJ-related genes or proteins. These results suggested that the intestinal physical barrier of the metabolic syndrome mice suffered from worsened damage after exposure to TiO_2_ NPs.

Researchers showed that overproduce inflammatory cytokines such as TNF-α, IL-1β, and IFN-γ could decrease the amounts of TJ-related proteins [38]. The intestinal inflammatory response was assessed, in the normal mice, and a slight inflammation was observed after exposure TiO_2_ NPs, and this discovery was similar with Mu et al. [20]. Moreover, the apoptosis-related genes under a high expression level, which also have closely connected to the TJ barrier homeostasis [39]. These results suggested that TiO_2_ NPs triggered severer immune inflammatory response in metabolic syndrome mice’s intestine, which induced subsequent worse intestine and liver injury. Toxic effects came from TiO_2_ NPs-induced ROS in intestinal epithelial cell was confirmed by previous study [40]. Furthermore, we explored the gut oxidative stress effect of which probably was the initial reason stimulate inflammation. In our works, whatever TiO_2_ NPs and fructose exposure alone could cause mild oxidative stress. However, TiO_2_ NPs aggravated oxidative stress in metabolic syndrome mice, which possible because fructose exposure leads the intestines being subject to long-term low inflammation and oxidative stress response, and this semi-pathological state made mice more sensitive and more easily impacted by TiO_2_ NPs. Therefore, the fructose-induced metabolic syndrome mice’s intestine would suffer more damage after exposure TiO_2_ NPs than normal healthy mice.

## 5. Conclusions

In summary, the connections among the severe toxicities of exposure to TiO_2_ NPs in the liver and intestinal barrier were assessed in fructose-induced metabolic syndrome mice. No change was found after low-dose TiO_2_ NPs were administered. However, serious hepatic inflammation, fibrosis, and apoptosis were observed in high-dose intake of TiO_2_ NPs due to more drastic oxidative stress in the liver. Moreover, TiO_2_ NPs exacerbated the increase in intestinal permeability and inflammatory response through exacerbating oxidative stress, thereby leading to increased TiO_2_ NPs and LPS being transferred to the liver and aggravating hepatic injury in metabolic syndrome mice. These results suggested that the metabolic syndrome population should be given further attention in terms of the hazard of TiO_2_ NPs to their health.

## Figures and Tables

**Table 1:** Primers of genes for RT-qPCR.

Gene	Primer	Sequence (5′~3′)
*TNF-α*	Forward	CTGAACTTCGGGGTGATCGG
	Reverse	GGCTTGTCACTCGAATTTTGAGA
*IL-1β*	Forward	GCAACTGTTCCTGAACTCAACT
	Reverse	ATCTTTTGGGGTCCGTCAACT
*IL-6*	Forward	ACAAGAAAGACAAAGCCAGAGT
	Reverse	GGAAATTGGGGTAGGAAGGAC
*TLR-4*	Forward	CTGTATTCCCTCAGCACTCTTGATT
	Reverse	TGCTTCTGTTCCTTGACCCACT
*VEGFA*	Forward	AGCCAAATTGGGGTTGAGGGT
	Reverse	GGAGCAAAGGTCACGAAAGCAG
*TGF-β*	Forward	GTCACTGGAGTTGTACGGCA
	Reverse	TCATGTCATGGATGGTGCCC
*IFN-γ*	Forward	AGACAATCAGGCCATCAGCA
	Reverse	TGGACCTGTGGGTTGTTGAC
*Caspase3*	Forward	GGAGGCTGACTTCCTGTATGCTT
	Reverse	CCTGTTAACGCGAGTGAGAATG
*Caspase9*	Forward	AAGAAGACCGGAGTGCAATG
	Reverse	CATGACAGGATTATACAACCGC
*ZO-1*	Forward	GCCGCTAAGAGCACAGCAA
	Reverse	TCCCCACTCGAAAATGAGGA
*OCLN*	Forward	TTGAAAGTCCACCTCCTTACAGA
	Reverse	CCGGATAAAAAGAGTACGCTGG
*CLDN2*	Forward	CTGCCAGGATTCTCGAGCTA
	Reverse	CCCAAGTACAGAGCCTCTCC
*MLCK*	Forward	TGCTTCTGACATACGGAGTT
	Reverse	GACATTGAAAGAGGTGCTG
*GAPDH*	Forward	ATGTGTCCGTCGTGGATCTG
	Reverse	GCCGTATTCATTGTCATACCAGG

## Data Availability

The data presented in this study are available in this article.
